# Supplementary material for: WordCommentsAnalyzer: A windows software tool for qualitative research
Source: F1000Res. 2018 Sep 4;7:536. Originally published 2018 May 3. [Version 2] doi: 10.12688/f1000research.14819.2 (PMC6137411; doi:10.12688/f1000research.14819.2)
Supplement: Supplementary file 3 [file f1000research-7-17614-s0002.tgz › b2a79496-4ada-47c4-9682-a99ce867836d.zip!/Eight Iranian Health Journals/Archives of Rehabilitation_35.docx]

© Archives of Rehabilitation. This work is licensed under CC BY-NC 4.0 (https://creativecommons.org/licenses/by-nc/4.0/).

Volume & Issue: دوره 11 -

Title: تأثیر ماساژ بر رشد و تکامل شیرخواران

http://rehabilitationj.uswr.ac.ir/article-1-740-fa.html

هدف: اهمیت رشد و تکامل شیرخواران و تأثیر غیر قابل انکار آن بر سلامت نسل های آتی سبب می شود تا همواره محققین در پی شناسایی راهکارهایی باشند که موجب بهبود شاخص های رشدی و تکاملی گردد. این مطالعه با هدف بررسی تاثیر ماساژ بر شاخص های رشدی و حیطه های تکاملی شیرخواران طراحی و اجرا شد . روش بررسی: این مطالعه از نوع کارآزمایی بالینی با تخصیص تصادفی است که بر روی 92 شیرخوار مراجعه کننده به مرکز بهداشتی درمانی امام خمینی شهر قم در سال 1388 انجام گرفت. ابزار گردآوری داده‌ها شامل یک فرم اطلاعات دموگرافیک و شاخص های رشدی شامل: وزن، قد، دورسر، دورسینه و دور بازو بودند. جهت شاخص های تکاملی در سن دو ماهگی از چک لیست بررسی تکامل استفاده گردید و در سن چهار ماهگی از آزمون تکاملی "پرسشنامه سنین و مراحل" استفاده گردید. شیرخواران در دو گروه آزمایش و شاهد قرار گرفتند. در گروه آزمایش به مادر آموزش داده شد که هرشب قبل از خواب و هر بار 15 دقیقه به مدت دو ماه شیر خوار را به روش ماساژ سوئدی لمس نماید. سپس شاخص های رشدی و تکاملی در چهار ماهگی بررسی و با هم مقایسه گردیدند. یافته ها: هر دو گروه از نظر جنس، شاخصهای رشد و تکامل در ورود به مطالعه همسان بودند. بعد از مداخله، افزایش وزن (P<0/001) دور سینه(P<0/001)، دور بازو(P<0/001) در گروه آزمایش بیشتر از شاهد بود و اختلاف آماری معنی داری وجود داشت. افزایش دور سر و قد در گروه آزمایش بیشتر بود اما تفاوت معنی داری نداشت.از نظر حیطه های تکاملی در 4 ماهگی در حیطه حرکات درشت (P>0/03) و شناختی- اجتماعی (P<0/001) در گروه آزمایش تفاوت معنی دار بود و در حیطه ارتباطی ارزش اخباری بینابینی بود (P = 0/058)، ولی در حیطه حرکات ظریف و حل مسئله اختلاف معنی دار مشاهده نگردید. نتیجه گیری: ماساژ بر شاخص های رشد و تکامل شیرخواران موثر است.

Title: آیا شدت اضطراب در مادران کودکان مبتلا به فلج مغزی بالاتر از مادران کودکان عادی است؟

http://rehabilitationj.uswr.ac.ir/article-1-679-fa.html

هدف: تعیین شدت اضطراب در مادران دارای کودکان CP وارتباط آن با نوع و شدت ناتوانی کودک بود. روش بررسی: این تحقیق به روش توصیفی- تحلیلی انجام شد. به این منظور 43 نفر از مادران دارای کودک 6 ماه تا 8 ساله CP که در کلینیک صبا، وابسته به دانشگاه علوم بهزیستی و توانبخشی در تهران تحت توانبخشی بودند، به روش نمونه گیری ساده (در دسترس) به عنوان گروه مورد انتخاب شدند. پس از اخذ رضایت از مادران، پرسشنامه هایی حاوی سوالات مشخصات دموگرافیک و آزمون اضطراب کتل ، توسط آنان تکمیل گردید و بر اساس نمره حاصله وجود و شدت اضطراب در مادران مشخص شد. نوع CP توسط متخصص کودکان و شدت ناتوانی حرکتی توسط کارشناس کاردرمانی با استفاده از آزمونGMFM تعیین شد. تعداد 77 نفر از مادران کودکان طبیعی نیز به عنوان گروه کنترل پرسشنامه ها را تکمیل نمودند. یافته ها: بین اضطراب در دو گروه تفاوت معناداری به لحاظ آماری وجود نداشت (P=0/663) ولی نمره اضطراب مادران فلج مغزی با توجه به نوع فلج مغزی اختلاف معنی داری را نشان داد (P=0/023). هم چنین رابطه معناداری بین شدت ناتوانی حرکتی (R=0/386)، وضعیت اقتصادی خانواده، سن مادران، جنسیت کودکان، تعداد افراد ساکن در منزل، نحوه ی تولد کودک، نسبت فامیلی بین والدین و سطح تحصیلات والدین، با میزان اضطراب مادران وجود نداشت. نتیجه گیری: با توجه به یافته ها و محدودیت های پژوهش به نظر می آید که نیاز به انجام پژوهش های بعدی ضروری است.

Title: پاسخ های عضلانی برانگیخته دهلیزی (VEMP) در کودکان

http://rehabilitationj.uswr.ac.ir/article-1-689-fa.html

هدف: پتانسیل های عضلانی برانگیخته دهلیزی (VEMP)، یکی از ابزارهای جدید برای بررسی عملکرد دهلیزی می باشند. علیرغم تعدّد تحقیقات انجام گرفته مرتبط با VEMP در بزرگسالان، تحقیقات اندکی در کودکان انجام گرفته است و ویژگی های پاسخ VEMP در کودکان کمتر بررسی شده است. هدف از این مطالعه، به دست آوردن مقادیر VEMP در این گروه سنی و مقایسه آن با بزرگسالان هنجار بود. روش بررسی: در این مطالعه مقطعی-تحلیلی، 19 کودک ( 10 پسر و 9 دختر) با محدوده سنی 12-3 سال شرکت کردند. از کودکان خواسته شد تا روی تخت خوابیده و برای انقباض عضله استرنوکلیدوماستوئید هر طرف، سر خود را 30 درجه به بالا خم کرده و به سمت مخالف آن عضله بچرخانند. پاسخ VEMP با استفاده از محرک تون برست 500 هرتز ثبت گردید. زمان نهفتگی امواج p13 و n23، آستانه و نسبت دامنه ای پاسخ، محاسبه شده و با نتایج به دست آمده در بزرگسالان مقایسه گردید. یافته ها: پاسخ های VEMP با محرک تون برست 500 هرتز در تمامی کودکان ثبت شد. مقایسه زمان نهفتگی، آستانه و نسبت دامنه ای پاسخ، هیچ گونه تفاوت معناداری بین کودکان و گروه کنترل (بزرگسالان) نشان نداد. نتیجه گیری: آزمون VEMP می تواند به عنوان یک آزمون غیر تهاجمی و ارزشمند، برای بررسی عملکرد دهلیزی و به خصوص رفلکس گردنی- دهلیزی در کودکان مورد استفاده قرار گیرد.

Title: بررسی تاثیر رویکرد آموزش نمونشی در بهبود عملکرد حرکتی درشت کودکان فلج مغزی اسپاستیک 3 تا 6 ساله در موسسه توانبخشی ولیعصر (عج) تهران

http://rehabilitationj.uswr.ac.ir/article-1-658-fa.html

هدف: فلج مغزی اختلال ی غیر پیشرونده در وضعیت و حرکت بدن می باشد که در اثر علل مختلفی ایجاد می شود. رویکردهای توانبخشی مختلفی در این کودکان از جمله، رویکرد آموزش نمونشی به کار می رود. مطالعه حاضر با هدف بررسی تاثیر رویکرد آموزش نمونشی در بهبود عملکرد حرکتی درشت کودکان فلج مغزی اسپاستیک 3 تا 6 ساله با هوشبهر طبیعی انجام شد. روش بررسی: این مطالعه از نوع شبه تجربی و مداخله ای (قبل و بعد) بود که روی 20 کودک فلج مغزی اسپاستیک 3 تا 6 ساله­ی در دسترس که شرایط ورود به مداخله را دارا بودند انجام شد. به منظور تعیین میزان هوشبهر کودکان از مقیاس هوشی وکسلر، و برای تعیین عملکرد حرکتی درشت قبل وبعد از مداخله از آزمون نرم افزاری GMFM-66 استفاده شد رویکرد آموزش نمونشی در یک برنامه 36جلسه ای (5 روز در هفته و هر جلسه روزانه 2 ساعت) برای کودکان انجام شد. یافته‌­ها: اختلاف معنی ­داری در عملکرد حرکتی درشت بعد از انجام مداخله درمانی دیده شد (P=0/003). ارزیابی وضعیت قبل و بعد در هر یک از سطوح عملکردی نشان داد که بیشترین تاثیر آموزش نمونشی، در آزمودنیهای سطح III می با شد . مقایسه سطوح از لحاظ عملکرد حرکات درشت نتیجه معنی داری مبنی بر تفاوت آنها نشان نداد(P=0/466). نتیجه‌گیری: رویکرد آموزش نمونشی باعث بهبود عملکرد حرکتی درشت می­گردد و در تمامی سطوح عملکردی حرکات درشت کاربرد دارد.

Title: مقایسه درک ساخت‌های مشتق از حرکت نحوی بین کودکان آسیب‌دیده شنوایی با کودکان شنوا

http://rehabilitationj.uswr.ac.ir/article-1-700-fa.html

هدف: آسیب شنوایی در دوران کودکی معمولاً بر همه جنبه‌های تولید و درک گفتار تأثیرگذار است. به نظر می‌رسد افرادآسیب‌دیده شنوایی از برخی مهارت‌های گفتاری و زبانی از جمله درک ساخت‌های مشتق از حرکت نحوی محروم هستند. هدف مطالعه حاضر مقایسه درک ساخت‌های مشتق از حرکت نحوی اسم بین کودکان آسیب‌دیده شنوایی و کودکان شنوای فارسی زبان می‌باشد. روش بررسی: این مطالعه از نوع مورد شاهدی بود که در آن 20 کودک شنوای 6-7 ساله و 20 کودک آسیب‌دیده شنوایی 8-12 ساله به روش نمونه‌گیری تصادفی ساده از میان مدارس عادی دولتی و مدارس استثنایی دولتی ویژه ناشنوایان مقطع ابتدایی انتخاب شدند. به جز کم‌شنوایی در دانش آموزان ناشنوا، افراد مورد مطالعه هیچ گونه بیماری یا مشکل عصبی تایید شده‌ای نداشتند. برای تعیین میزان و نوع کم شنوایی، به پرونده وضعیت شنوایی دانش‌آموزان و تأیید شنوایی‌شناس مدارس ناشنوایان استناد شد. برای انجام آزمون درک، از تکلیف محقق ساخته انطباق جمله – تصویر استفاده شد. در ابتدا روایی محتوایی احراز شد و بررسی پایایی آن نیز با ضریب آلفای کرونباخ %77 مورد تأیید قرار گرفت. داده‌ها با استفاده از آزمون‌های آماری تی‌مستقل و یو-‌من ویتنی و نرم‌افزار SPSS مورد تجزیه و تحلیل قرار گرفت . یافته‌ها: بین نمره درک ساخت‌های مشتق از حرکت نحوی، در کودکان شنوا و آسیب‌دیده شنوایی تفاوت معناداری وجود داشت (P<0/05)درک کودکان آسیب‌دیده شنوایی از جمله‌های دارای ساخت متعارف بهتر از جمله‌های مشتق از حرکت نحوی بود. نتیجه‌گیری: پاسخ‌های نادرست کودکان آسیب‌دیده شنوایی به تکلیف انطباق جمله – تصویر، بیانگر درک ناکامل آنها از جمله‌های مشتق از حرکت نحوی است و تفاوت چشمگیر کودکان شنوا و آسیب‌دیده شنوایی در درک جمله‌های مشتق از حرکت نحوی نشانگر نیاز ویژه این گروه به آموزش چنین ساخت‌هایی است.

Title: بررسی ارتباط حاملگی پر خطر با تأخیر تکاملی کودکان 60-4 ماهه شهر اصفهان سال89-1388

http://rehabilitationj.uswr.ac.ir/article-1-695-fa.html

هدف: پیشرفت در علم پزشکی و موفقیت در افزایش میزان بقای شیرخواران آسیب پذیر مسئله تکامل آتی این کودکان را مطرح کرده است. خطراتی که سلامت کودک را تهدید می کند گاهی از دوران جنینی شروع میشود. این مطالعه با هدف همبستگی حاملگی پر خطر با تأخیر تکاملی کودکان 60-4 ماهه مراجعه کننده به مراکز بهداشتی درمانی وابسته به دانشگاه علوم پزشکی و خدمات بهداشتی درمانی اصفهان سال 1388 انجام گرفت. روش بررسی: این مطالعه به شیوه توصیفی بر روی 401 مادر و کودک 60-4 ماهه آنها مراجعه کننده به مراکز بهداشتی درمانی وابسته به دانشگاه علوم پزشکی و خدمات بهداشتی درمانی اصفهان سال 1388 به روش نمونه گیری چند مرحله ای انجام شد. ابزار گردآوری داده ها شامل فرم اطلاعاتی، چک لیست جمع آوری کننده اطلاعات و آزمون تکاملی "پرسشنامه های سنین و مراحل" بود و اندازه گیری های تن سنجی کودک توسط پژوهشگر با متر و ترازو گرفته شد. سپس داده ها با نرم افزار spss 18 و آزمونهای آماری مجذور کای، T مستقل، من ویتنی و رگرسیون لجستیک مورد تجزیه و تحلیل آماری قرار گرفتند. یافته‌ها: نتایج نشان داد میانگین سنی کودکان در گروه حاملگی کم خطر 16±22 ماه و در گروه حاملگی پرخطر 14.8±18.9 ماه و بیشترین فراوانی جنسیت دختر می باشند. میانگین سنی مادران در حاملگی کم خطر 3.1±26.31 سال و در حاملگی پرخطر 4.7±27.56 سال و بیشترین فراوانی مادران با تحصیلات دبیرستان و شغل خانه دار می باشد. شیوع حاملگی پرخطر در جامعه مورد نظر %92 و شیوع تأخیر تکاملی کودکان %18.7 می باشد. چندقلویی، وزن کم بدو تولد، سقط تکراری، دیابت و اختلالات طبی دوران بارداری همبستگی معناداری با تأخیر تکاملی کودکان داشتند(P<0/04) و در مدل لجستیک متغیرهای جنسیت پسر، وزن کم بدو تولد، ازدواج فامیلی و اختلالات طبی بارداری همبستگی معناداری با تأخیر تکاملی کودکان نشان دادند(P<0/05). همچنین شاخص توده بدنی مادر و وضعیت اقتصادی و اجتماعی مقدار احتمال نزدیک به سطح معناداری داشته اند (P=0/05)و سایر متغیرهای حاملگی پرخطر همبستگی نداشتند (P>0/05). حاملگی پرخطر همبستگی معناداری با تأخیر تکاملی کودکان و حیطه های آن نشان نداد (p=0/06). نتیجه‌گیری: این مطالعه نشان داد که احتمال بروز تأخیر تکاملی کودکان به دلیل حاملگی پرخطر کم می باشد، لیکن برخی متغیرهای حاملگی پرخطر همبستگی معناداری با تأخیر تکاملی کودکان داشتند. لزوم تحقیقات بیشتر با توجه به عوامل بیولوژیک، مادری و محیطی توصیه می گردد.

Title: تاثیر آموزش هنرهای تجسمی بر افزایش مهارتهای ادراک بینایی دانش آموزان حساب نارسا

http://rehabilitationj.uswr.ac.ir/article-1-706-fa.html

هدف: پژوهش حاضرباهدف بررسی تأثیر آموزش هنرهای تجسمی بر افزایش مهارتهای ادراک بینایی دانش آموزان حساب نارسا انجام شد . روش بررسی: پژوهش کاربردی و روش ازنوع نیمه آزمایشی پیش آزمون و پس آزمون با گروه کنترل بود وروش نمونه گیری به صورت غربالگری ونمونه شامل 30 نفر از دانش آموزان حساب نارسای پسر پایه های دوم و سوم دبستان بود وابزار مورد استفاده عبارت بودند ازپرسشنامه تشخیصی حساب نارسایی DSM-IV ،آزمون پیشرفته ادراک بینایی فراستیگ وآزمون ریاضی ایران –کی مت. آزمودنیها پس از پیش آزمون درسه گروه 10نفره نقاشی،سفال و کنترل قرارداده شدندبه طوری که به یک گروه نقاشی وبه یک گروه سفال آموزش داده شد و به گروه کنترل آموزشی داده نشد و بعد از آموزش از هر سه گروه پس آزمون بعمل آمد. یافته‌ها: تحلیل داده های پژوهش با استفاده از روشهای آمار توصیفی(شاخص های گرایش مرکزی) و مدل آماری تحلیل کواریانس تفاوت معنا داری را بین سه گروه نقاشی،سفال وکنترل نشان داد. نتیجه گیری: آموزش هنرهای تجسمی بر مهارتهای ادراک بینایی به غیر از درک ثبات شکل موثر است.

Title: تأثیر محدودیت درمانی تعدیل یافته بر کیفیت گرفتن در کودکان فلج مغزی نیمه بدن

http://rehabilitationj.uswr.ac.ir/article-1-697-fa.html

هدف: محدودیت درمانی روشی است که در آن با محدود کردن اندام سالم فردی که دچار فلج نیمه بدن(همی پلژی) شده، اجبار در استفاده از اندام مبتلا را فراهم می آورد. به دلیل برخی نگرانی ها روش تعدیل یافته آن گسترش یافت و هدف این پژوهش ، بررسی تاثیر استفاده از محدودیت درمانی تعدیل یافته بر کیفیت گرفتن در کودکان فلج مغزی نیمه بدن است. روش بررسی: در این مطالعه تجربی و بالینی 21 کودک در دسترس با توجه به معیارهای ورود به مطالعه انتخاب شدند و به طور تصادفی تعادلی به دو گروه آزمایش و کنترل تقسیم شدند. تمرینات متداول کاردرمانی در طی 6 هفته برای هر دو گروه به طور یکسان انجام شد و گروه مداخله روزانه به مدت 3 ساعت ار روش محدودیت درمانی تعدیل یافته نیز استفاده کردند. کیفیت گرفتن قبل و پس از مداخله بر اساس آزمون کیفیت مهارت های اندام فوقانی (ًQUEST) ارزیابی گردید.داده های حاصل با استفاده از آزمون های آماری کولموگروف- اسمیرنوف،تی- مستقل و اندازه گیری داده های تکراریکه به صورت هر دو هفته یکبار مورد تحلیل قرار می گرفت، انجام شد. یافته‌ها: 11 کودک در گروه آزمایش (7 دختر،4 پسر) با میانگین سنی (47/17±55/46) ماه و 10 کودک در گروه شاهد (5 دختر،5 پسر) با میانگین سنی(19/19±10/48) ماه مورد بررسی قرار گرفتند. آنالیز های آماری بین گروهی با استفاده از آزمون تی مستقل تفاوت معناداری بین کیفیت گرفتن بعد از شش هفته مداخله نشان داد (P>0/05) و آنالیز آماری با استفاده از آزمون اندازه گیری های تکراری معناداری را در 2 هفته بعد از مداخله و دو هفته دوم نشان داد (P=0/001) و در دو هفته سوم معنادار نبود (P>0/05). نتیجه گیری: نتایج این مطالعه نشان داد که محدودیت درمانی تعدیل یافته بر کیفیت گرفتن کودکان فلج مغزی نیمه بدن تأثیر دارد مطالعات بیشتر به منظور رشد و تحکیم بخشیدن به این روش را پیشنهاد می کنیم.

Title: بررسی تاثیر برنامه تحریکات دهانی بکمن بر زمان دستیابی به تغذیه دهانی مستقل نوزادان نارس بستری در واحد مراقبتهای ویژه نوزادان

http://rehabilitationj.uswr.ac.ir/article-1-717-fa.html

هدف: هدف این مقاله بررسی تاثیر برنامه تحریکات دهانی بکمن بر زمان دستیابی به تغذیه دهانی مستقل در نوزادان نارس متولد شده با سن جنینی (32-26) هفته می باشد. روش بررسی: مطالعه حاضر از نوع کارآزمایی بالینی می باشد.آزمودنیهای این ﭘﮊ وهش شامل 20 نوزادان نارس (32-26 هفته) بخش مراقبت های ویژه نوزادان بیمارستان ولیعصر تهران می باشند که به صورت تخصیص تصادفی در دو گروه آزمایش و کنترل قرار گرفتند. نوزادان گروه آزمایش برنامه تحریک دهانی شامل 15 دقیقه تحریک که 12 دقیقه از آن به تحریک اطراف و داخل دهان و 3 دقیقه پایانی آن به مکیدن غیر تغذیه ای (مکیدن انگشت ﭘﮊ وهشگر ) اختصاص می یافت را دریافت می کردند که این برنامه تحریکی به مدت 10 روز متوالی انجام می شد ، نوزادان گروه کنترل برنامه تحریکات دهانی را در یافت نمی کردند.   گروه مورد و شاهد از لحاظ زمان دستیابی به یک، چهار و هشت بار تغذیه دهانی در روز ، فاصله زمانی بین دفعات مختلف تغذیه و مدت زمان بستری در بیمارستان مورد بررسی و مقایسه قرار گرفتند. یافته‌ها: نوزادان گروه آزمایش نسبت به گروه کنترل، تغذیه دهانی مستقل را بطور معناداری زودتر کسب کردند (P<0/001) . مدت زمان بستری در بیمارستان در گروه آزمایش بطور معنی داری ، کوتاه تر از گروه کنترل بود (P<0/001) . نتیجه‌گیری: نتایج این مطالعه از این فرضیه حمایت می کند که تحریکات دهانی پیش تغذیه ای و مکیدن غیر تغذیه ای زمان دستیابی به تغذیه دهانی مستقل وزمان ترخیص از بیمارستان را در نوزادان نارس تسریع می بخشند .

Title: بررسی اثربخشی تقویت کننده پته‌ای بر پیشرفت تحصیلی در درس علوم تجربی دانش آموزان پسر کم توان ذهنی پایه سوم راهنمایی

http://rehabilitationj.uswr.ac.ir/article-1-809-fa.html

هدف: این پژوهش اثربخشی تقویت کننده پته‌ای بر پیشرفت تحصیلی در درس علوم تجربی دانش آموزان پسر کم توان ذهنی پایه سوم راهنمایی در شهرستان‌های استان تهران را بررسی می‌کند. روش بررسی: پژوهش حاضر یک مطالعه آزمایشی با طرح پیش آزمون- پس آزمون با گروه کنترل است، که به صورت خوشه‌ای، نمونه‌ای به حجم 20 نفر از دانش آموزان کم توان ذهنی پایه سوم راهنمایی انتخاب شد، پس از انتساب تصادفی دانش آموزان در دو گروه آزمایش و کنترل، برای دانش آموزان گروه آزمایشی طی 17 جلسه آموزش از تقویت کننده پته‌ای استفاده شد و به دانش آموزان گروه کنترل تقویت کننده‌ای ارائه نشد. ابزار جمع آوری اطلاعات آزمون هوش وکسلر (برای همتا کردن گروه‌ها به لحاظ بهره هوشی) و آزمون‌های معلم ساخته پیشرفت تحصیلی در درس علوم تجربی بود. داده‌های به دست آمده با استفاده از آمار توصیفی (میانگین، انحراف معیار، نمودار و جدول فراوانی) و آزمون تی همبسته تجزیه و تحلیل شد. یافته‌ها: نتایج نشان داد که میانگین نمرات پیشرفت تحصیلی درس علوم تجربی گروه تقویت پته‌ای به طور معناداری بیشتر از گروه کنترل (P<0/001) است. این مداخله باعث افزایش میانگین نمرات گروه آزمایش در درس علوم تجربی نسبت به گروه کنترل شد. نتیجه گیری: تقویت پته‌ای باعث پیشرفت تحصیلی دانش آموزان کم توان ذهنی پایه سوم راهنمایی در درس علوم تجربی می‌شود، بنابراین اثر تقویت پته‌ای بر پیشرفت تحصیلی در درس علوم تجربی بیشتر از گروه کنترل است، کاربرد تقویت کننده‌های پته‌ای انگیزه پیشرفت در دانش آموزان کم توان ذهنی را افزایش می‌دهد.

Title: تأثیر نقاشی بر کاهش پرخاشگری دانش آموزان دختر عقب مانده ذهنی آموزش پذیر مقطع ابتدایی

http://rehabilitationj.uswr.ac.ir/article-1-391-fa.html

هدف: رفتار پرخاشگری مشکلی متداول میان افراد عقب مانده ی ذهنی است. یکی از روش های پیشنهاد شده جهت درمان پرخاشگری، هنردرمانی است. بر این اساس، هدف از مطالعه حاضر، تعیین میزان تأثیر نقاشی برکاهش پرخاشگری دانش آموزان دختر عقب مانده ذهنی آموزش پذیر مقطع ابتدایی شهرستان کرج می باشد. روش بررسی: در این پژوهش که یک مطالعه مداخله ای از نوع شبه تجربی با طرح پیش آزمون - پس آزمون با گروه های کنترل و آزمایش است، 52 دانش آموز دختر عقب مانده ذهنی آموزش پذیر مقطع ابتدایی شهرستان کرج با استفاده از روش نمونه گیری خوشه ای چند مرحله ای پس از لحاظ کردن ملاک های ورود به مطالعه، جهت شرکت در پژوهش انتخاب شدند. برای سنجش میزان پرخاشگری دانش آموزان از پرسشنامه پرخاشگری کودکان کم توان ذهنی (.B.D.Gh) استفاده شد. پس از استخراج نتایج پیش آزمون، با توجه به پایه ی تحصیلی، سن و میزان پرخاشگری، دانش آموزان در دو گروه همتا قرار داده شدند و به صورت تصادفی یک گروه به عنوان گروه کنترل و دیگری به عنوان گروه آزمایش انتخاب شد. سپس گروه آزمایش در 8 جلسه ی پی در پی، هر روز طبق دستورالعمل به نقاشی پرداختند. اجرای پرسشنامه پرخاشگری به عنوان پس آزمون، 10 روز پس از آخرین جلسه اجرا انجام شد تا مشخص شود که آیا این روش در کاهش پرخاشگری مؤثر بوده است یا خیر؟ داده های بدست آمده با استفاده از آزمون های آماری تی مستقل و زوجی، کولموگروف اسمیرنوف، تحلیل واریانس یک راهه و آزمون تعقیبی توکی مورد تجزیه و تحلیل قرار گرفتند. یافته ها: نتایج پژوهش نشان داد که بین میانگین نمرات پیش آزمون و پس آزمون پرخاشگری کل، پرخاشگری غیر کلامی به خود، پرخاشگری کلامی به غیر خود و پرخاشگری غیر کلامی به غیر خود گروه آزمایش در مقایسه با گروه کنترل تفاوت معناداری وجود دارد (001/0>P). ولی بین میانگین نمرات پیش آزمون و پس آزمون پرخاشگری کلامی به خود گروه آزمایش در مقایسه با گروه کنترل تفاوت معناداری وجود ندارد. نتیجه گیری: نقاشی روش مؤثری برای کاهش پرخاشگری دانش آموزان دختر عقب مانده ذهنی آموزش پذیر مقطع ابتدایی است.

Title: تأثیر نمایش عروسکی(همراه با نور uv)در کاهش پرخاشگری دانش آموزان عقب مانده ذهنی آموزش پذیر

http://rehabilitationj.uswr.ac.ir/article-1-399-fa.html

مقدمه: رفتارهای ضد اجتماعی و پرخاشگری در زمره شایع ترین علل ارجاع کودکان و نوجوانان به کلینیکهای بهداشت روانی هستند. که در بین افراد عقب مانده ذهنی فراوان به چشم می خورد. جهت درمان و کاهش پرخاشگری کودکان کم توان ذهنی روش های مختلفی پیشنهاد شده است. نمایش عروسکی به عنوان یکی از این مداخلات، می تواند در درمان پرخاشگری و مشکلات رفتاری کودکان عقب مانده ذهنی موثر باشد. هدف از مطالعه حاضر، تعیین میزان تأثیر نمایش عروسکی(همراه با نور uv ) در کاهش پرخاشگری دانش آموزان عقب مانده ذهنی آموزش پذیر مقطع ابتدایی می باشد.  روش بررسی: این پژوهش یک مطالعه شبه تجربی است. در این مطالعه 40 دانش آموز عقب مانده ذهنی به روش تصادفی ساده از بین مدارس تهران انتخاب و به صورت تصادفی در دو گروه آزمایش (20 نفر)، و گروه کنترل (20 نفر) قرار گرفتند. برای گروه آزمایش هفت جلسه پی در پی (15 الی 20 دقیقه) نمایش عروسکی همراه با نور ( uv )، بر گزار گردید و گروه کنترل در جریان برنامه های عادی مدرسه قرار گرفتند. ابزار گرد آوری داده ها شامل پرسشنامه پرخاشگری کودکان کم توان ذهنی ( B.D.GH ) بود. تجزیه و تحلیل آماری به وسیله آزمون های آماری تی زوجی و تی مستقل انجام شد.  یافته ها: میانگین پرخاشگری گروه کنترل و آزمایش در پیش آزمون پرخاشگری تفاوت آماری معناداری نداشته (P=0/999) ولی در پس آزمون، میانگین پرخاشگری گروه آزمایش به طور معناداری کمتر از گروه کنترل است (p<0/001). همچنین میانگین پرخاشگری گروه آزمایش در پس آزمون به طور معناداری کمتر از پیش آزمون است (p<0/001). این نشان می دهد آزمایش باعث کاهش پرخاشگری گروه آزمایش در پس آزمون شده است. ولی میانگین پرخاشگری گروه کنترل در پس آزمون تفاوت معناداری با پیش آزمون ندارد .  نتیجه گیری: یافته های این مطالعه نشان داد که آزمایش اثر گذار بوده و نمایش عروسکی باعث کاهش پرخاشگری دانش آموزان عقب مانده ذهنی شده است.

Title: مقایسه ویژگی‌های جمعیت‌شناختی و زیستی والدین کودکان بیش از یک فرزند استثنایی با والدین کودکان عادی

http://rehabilitationj.uswr.ac.ir/article-1-566-fa.html

هدف: از پژوهش حاضر دست یابی به علل و عوامل احتمالی موثر در تولد کودکان استثنایی در خانواده هایی با بیش از یک فرزند استثنایی است که از طریق تحلیل ویژگیهای والدین با بیش از یک فرزند استثنایی در دو بخش ویژگیهای جمعیت‌شناختی و زیستی و مقایسه آنها با والدین دارای یک فرزند استثنایی و والدین دارای فرزندان عادی در جامعه مورد مطالعه (شهرستان ارومیه) صورت گرفت.  روش بررسی: پژوهش از نوع مقایسه ای پس رویدادی است و جامعه آماری شامل کلیه والدین با بیش از یک فرزند استثنایی و والدین با فرزند عادی در شهرستان ارومیه می باشد . نمونه آماری شامل ۸۰ زوج از والدین با بیش از یک فرزند استثنایی و ۵۳ زوج از والدین با فرزند عادی می باشد . در پژوهش حاضر از ابزار پرسشنامه ویژگیهای زیستی- شناختی والدین با بیش از یک فرزند استثنایی و پرسشنامه سلامت عمومی استفاده گردید. برای تحلیل داده‌ها از آزمون‌های مجذور خی توافقی، یو من ویتنی و آزمون تی استفاده شد. یافته‌ها: در متغیرهای جمعیت شناختی کودک نیز در متغیرهای ترتیب تولد کودک (p<0/001). سابقه ابتلا به بیماری (0/006=P)، نوع نارسایی کودک (0/001) و علت اصلی معلولیت (0/014=P)در بین دو گروه تفاوت معنادار بود. در مورد متغیرهای زیستی، نوع ازدواج والدین (0/019)، سن پدر و مادر هنگام ازدواج (0/001>P)، سن مادر در دوره بارداری (0/031=P)، محل تولد پدر و مادر (p<0/001). سلامت عمومی مادر (0/012=P) و گروه خونی پدر و مادر (p<0/001). تفاوت معناداری مشاهده شد. نتیجه‌گیری: پیشگیری و برنامه‌ریزی برای کنترل متغیرهای مؤثر بر میزان تولد کودکان استثنایی در خانواده‌ها که در تحقیق حاضر بررسی شده است، به کاهش معلولیت‌ها کمک خواهد کرد.
